# Optimising conditions and environments for digital participation in later life: A macro-meso-micro framework of partnership-building

**DOI:** 10.3389/fpsyg.2023.1107024

**Published:** 2023-03-02

**Authors:** Arlind Reuter, Wenqian Xu, Susanne Iwarsson, Tobias Olsson, Steven M. Schmidt

**Affiliations:** ^1^Department of Health Sciences, Faculty of Medicine, Lund University, Lund, Sweden; ^2^Department of Culture, Languages and Media, Faculty of Education and Society, Malmö University, Malmö, Sweden

**Keywords:** community development, digital participation, environments of ageing, multistakeholder and multisector collaboration, partnership building

## Abstract

The ongoing digitalisation of societies, exacerbated by the COVID-19 pandemic, has led to increased efforts to ensure the digital inclusion of older adults. Digital inclusion strategies throughout the COVID-19 pandemic predominantly focused on increasing access and basic digital literacy of Information and Communication Technologies (ICTs) for all members of society. Older adults, who are more likely to experience digital exclusion, are amongst the target groups of digital inclusion strategies. We propose that beyond digital inclusion, there is a need to focus on digital participation and optimise opportunities for everyone to participate in communities and society in post-pandemic times. Creative digital skills are the foundation of digital participation and can lead to a variety of contributions. Digital participation offers conditions that support agency and active contributions in a digitalised society. Taking macro-, meso-, and micro-level enablers of digital participation in later life into account, we argue for the establishment and implementation of multi-layered and multisectoral partnerships that address environmental factors (including social and physical dimensions) of digital participation and create opportunities for diverse, meaningful and fulfilling engagement with ICTs in later life. The partnership approach can be used in designing and implementing digital participation programmes and should be further evaluated against the needs and lived experiences of older individuals. Foresighted research is needed to investigate key factors of effective partnerships for optimising environments for digital participation in later life.

## 1. Introduction

The intersecting trends of population ageing and digitalisation have resulted in a focus on digital technology and later life. In highly digitalised countries such as Sweden, there is a high use of Information and Communication Technologies (ICTs) amongst older age groups. About 80% of people aged 66 years and over in Sweden are Internet users (The Swedes and the Internet, 2021). In many countries, digital inclusion programmes have been offered to older adults (Davidson, 2018; Hunsaker and Hargittai, 2018; Olsson et al., 2019; United Nations, 2020). The primary goal of digital inclusion programmes is to improve the accessibility of digital public services (Davidson, 2018). Despite the availability of inclusion programmes, experiences of digital technology in later life may differ significantly based on age, gender, race/ethnicity, geographic location, socioeconomic status, lifestyle and their intersecting effects (Hunsaker and Hargittai, 2018). Older adults are a heterogenous group in the digital world with various understandings of digital technology and different levels of knowledge, skills, abilities, and resources for digital technology use. However, research on digital inclusion in later life has not adequately reflected the manifold ways in which older adults experience digital technologies (Vines et al., 2015; Reuter et al., 2020b). To improve our understanding of the diverse use of digital technologies in later life, individual needs, preferences, and concerns in a digitalised society need to be recognised and different ways of and conditions for digital participation in later life should be investigated.

During the COVID-19 pandemic, community-based participation shifted from in-person activities towards digital spaces (Pantić et al., 2021). Public health measures, such as lockdowns, accelerated the digitalisation of civic activities (Budd et al., 2020; Mao et al., 2021). This development towards a digitalised civic life posed challenges to older individuals who wished to remain civically active. It also highlighted limitations of a digital inclusion approach, which in its current form focuses on digital accessibility, affordability, and literacy, rather than on the creative skills needed to support older adults’ active involvement and contributions in digitalised society. Whilst digital inclusion is one of the foundations for civic participation and social inclusion in later life (Milenkova and Lendzhova, 2021), active digital participation in later life remains under-explored (Serrat et al., 2020). Digital participation focuses on active involvement in digital society through the use of ICTs, with digital inclusion and accessibility merely representing two elements of the concept (Seifert and Rössel, 2022). Creative digital skills, such as skills to create digital content, are needed to achieve active involvement. The concept of digital participation acknowledges digital inequities and whether older adults participate actively or passively in digital society depending on usage, skills, social support, and self-perceptions (Seifert and Rössel, 2022). Indeed, participatory digital skills have become more important throughout the COVID-19 pandemic due to the shift from in-person participation into digital spaces. Expanding debates on digitalisation and ageing towards a focus on digital participation is an opportunity to further improve the lives of older adults in a digital society.

In the post-pandemic context, the digital lives of older adults may be improved using two approaches. The first approach is to continue to tackle digital inequities by promoting digital inclusion with the goal to improve digital access and digital literacy, which may be prerequisites for social inclusion in some contexts. This encompasses the continuous provision of accessible and affordable technology, and the training of basic digital skills required to navigate relevant services safely [United Nations Economic Commission for Europe (UNECE), 2021] The second approach is to create inclusive and diverse opportunities for older adults to participate digitally in communities and society. This encompasses creating age-friendly environments that support and encourage creative and active contributions, for example supporting the creation of digital content such as blogs or podcasts. Digital participation links closely to citizenship and the skills needed to take part in civic activities online, for example advocating for community matters or contributing to petitions. Recognising that the (non-)use of digital technology can be a conscious, individual and active choice (e.g., Waycott et al., 2016), not using digital technology should not be an obstacle to accessing basic services. Thus, maintaining conventional non-digital methods and services is vital to social inclusion.

Against this background, we pinpoint important enablers for digital participation in later life. An enabler is “something or someone that makes it possible for a particular thing to happen or be done” (Cambridge Dictionary, 2023). The purpose of this perspective article is not to generate a comprehensive overview of all enablers, but to expand current academic thinking beyond digital inclusion and literacy topics. The perspectives outlined here can contribute to prioritising digital participation in ageing policy and research agendas, as well as showcase opportunities for diverse, meaningful and fulfilling digital lives of older adults beyond the COVID-19 pandemic.

## 2. Macro-meso-micro level enablers to digital participation

We use the term ‘enabler’ broadly to describe a variety of individual, environmental, social, structural, and technological conditions that encourage digital participation in later life. The enablers outlined can be of international relevance, given digital exclusion and inequity in later life as common challenges across countries (e.g., in Europe see Esteban-Navarro et al., 2020; in the US see Yu et al., 2016; in Asia see Liu et al., 2021). Based on the enablers we identified, we propose a macro-meso-micro framework of partnership building, and further highlight the importance of mobilising and leveraging partnership resources, as well as utilising knowledge for action on different levels.

### 2.1. Macro level

*Policy* can shape most determinants of healthy ageing (World Health Organization, 2020), such as digital inclusion and equity. The focus of many policy and advocacy initiatives before and during the COVID-19 pandemic was on tackling digital exclusion and addressing digital disadvantages. Considering the continuous policy directions and improvements in enhancing digital inclusion, access and skills amongst older adults [e.g., for the progress made in Europe and North America, see United Nations Economic Commission for Europe (UNECE), 2022; for the progress made in Asia and the Pacific, see United Nations Economic and Social Commission for Asia and the Pacific (UNESCAP), 2022], more policy endeavours are needed to promote digital participation in later life. Existing digital policies should be reviewed and updated in consultations with older adults, their families, communities and other stakeholders in order to develop supportive policies for older adults to contribute to digitalised societies. This includes a stronger policy focus on digital participation skills beyond the existing digital inclusion policies. Given that multiple factors such as financial security and social support (Olsson et al., 2019) affect digital participation in later life (Hargittai et al., 2019), a participatory multisectoral approach is required to facilitate policy dialogue on the issues of digitalisation and ageing.

Creating a *positive culture* around older adults’ use of digital technologies is vital to meaningful digital participation in later life. Societal ageism has increased in many countries during the COVID-19 pandemic, reflected in prevalent notions of older adults as a “vulnerable group” (United Nations, 2020; Ayalon et al., 2021). Ageism manifests in social and cultural discourses of digital technology (Mannheim et al., 2022). Digital technology, such as artificial intelligence, can reproduce and generate new forms of ageism that affect digital participation and experience in later life (Rosales and Fernández-Ardèvol, 2020; Chu et al., 2022; World Health Organization, 2022). Ageism in digital technology can be self-directed, for example through negative self-perceptions and attitudes towards technology (Choi et al., 2020). Self-ageism is a barrier to technology adoption and engagement amongst older adults (Köttl et al., 2022). Ageism can lead to negative health and wellbeing outcomes and affect digital lives of older adults in households, communities and society. As indicated by a systematic review on digital inclusion programmes by Gates and Wilson-Menzfeld (2022), addressing ageist stereotypes, for example the perception of lower ability to engage with digital skills due to age, may avoid negative experiences in learning new digital skills and build confidence. It is important to consider individual needs and involve users across generations in the policies aiming to support digital participation (Fristedt et al., 2021). Tackling ageism can therefore contribute to a positive self or social awareness of ageing with digital technology and enable more older adults to become active, competent and confident digital citizens.

### 2.2. Meso level

*Knowledge and skills* in communities and civil society organisations are assets to digital participation and can potentially be leveraged in digital participation initiatives for older adults. Capacity building efforts are needed to increase digital knowledge and skills within digitally disadvantaged groups to sustain their digital participation. As an example of a digital participation initiative in Sweden, SeniorNet (a civil society organisation) set up study programmes to support advanced use of technology in later life. Moving from a user perspective towards a citizen perspective encourages positive changes older adults can generate in their living environments through the use of digital technologies. For older adults who are already online, digital communities can be further optimised to enhance wellbeing. Harley et al. (2014) proposed initiatives to improve digital participation, such as creating private “family rooms” and anonymous “sharing spaces” in online communities for older adults to connect online and local communities. In addition to utilising existing community resources, it is important to enhance community social capital and promote a participatory culture, thereby contributing to sustainability. As noted by Lu et al. (2022), community social capital can be enhanced with three approaches, namely promoting emotional meaningfulness, including older adults as co-producers of community activities, and cultivating an inclusive and equitable society. These approaches can be an inspiration in building up community capital and capacity for digital participation and therefore support active digital citizenship in later life.

*Established community development programmes* can be a window of opportunity to include digital participation initiatives. Age-friendly initiatives, such as the WHO’s age-friendly cities and communities (AFCC), which aim to increase civic participation in later life, are yet to incorporate digital environments in their policy frameworks (Marston and van Hoof, 2019). As a response, Liddle et al. (2020) proposed integrating digital environments into conceptual understandings of digitally connected AFCCs, with sensitivity to local contexts. Moving from a digital inclusion approach towards a digital participatory approach has the potential to support older adults in executing their digital citizenship to its fullest. This encompasses, for example, increasing digital participatory skills that support older adults in advocating for topics that matter to them on the local level (Clarke et al., 2016) and support their engagement and leadership in policy-and decision-making processes and interventions.

*Digitalised local organisations and services* pose challenges and opportunities to digital participation in later life. Digital technology that is not age-inclusive can challenge older adults’ use of digital services. In contrast, volunteering and other collective types of civic participation in later life can cultivate new digital participatory skills. In fact, extant research reveals positive correlations between digital competence and participation in civil society organisations (Olsson et al., 2019). Throughout the COVID-19 pandemic many community organisations shifted their engagement into digital spaces through virtual meetings. As this trend may continue and become part of the “new normal” in post-pandemic times, there is a need to support communities that (are willing to) address digital aspects of civic participation and boost creativity for new forms of digital and in-person engagement in later life. From a future-oriented perspective, older adults in increasingly digitalised and technology-mediated community spaces may dynamically choose to shift between different types of civic participation. Volunteering in its traditional sense is often focused on in-person interactions. Thus, digitalised volunteering, such as taking part in virtual neighbourhood meetings or volunteering to teach digital skills, offers opportunities for wider engagement beyond geographic boundaries or local issues. This in turn can tie into wider societal or political debates (Reuter, 2021).

### 2.3. Micro level

*Technology-mediated interactions* shape the process and experiences of digital participation and specifically equip older adults with the necessary skills to take on leading roles in digitalised civic activities (Trentham and Neysmith, 2018). Whilst on a micro-level the intergenerational family context plays an important role in the learning of digital skills (Martínez and Olsson, 2022), a long-term structure of support is vital to address diverse challenges that older individuals may face and improve their lived experiences and wellbeing (Manchester and Facer, 2015; Fischl et al., 2017, 2020), which may involve partner, children and grandchildren, close friends, and neighbours. Considering microenvironments of digital participation within and beyond the family context can also encompass peer-to-peer learning activities, in which older adults provide support to others in digital spaces (Hill et al., 2015). Addressing micro-level influencing factors of digital participation can help address older individuals’ needs, preferences and concerns. This may facilitate active, positive and sustainable learning experiences across the life course, which support individuals to dynamically adapt to emerging technologies. Community digital learning programmes should be tailored to meet the needs, preferences and expectations of older individuals and their families. Creating prerequisites for the development of older adults’ digital participatory skills and competence in creating, evaluating and communicating participation can help create alternative images of older adults, thus challenging societal ageism.

## 3. A proposed partnership framework to connect multi-level enablers in the community

**Figure 1:**
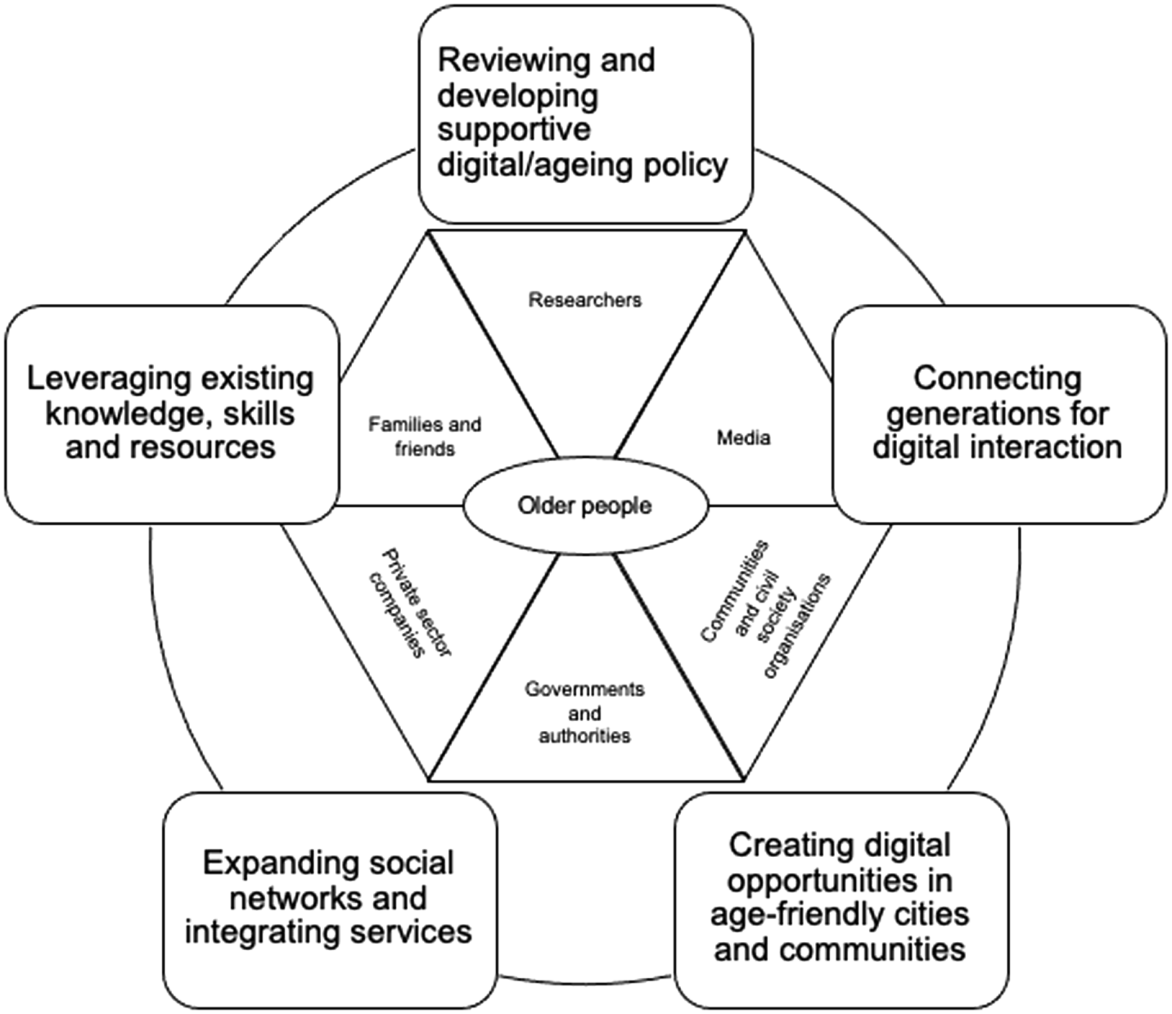
A visual guide to partnership building for digital participation in later life.

Building partnerships has long been recognised as an effective approach to promoting health and wellbeing on the community level (Israel et al., 2001). Digital participation in later life, however, has not been sufficiently addressed in its complexity of stakeholder engagement. The focus-to-date of multi-stakeholder partnership and action in promoting digital inclusion and participation in later life is often health-related. However, as argued above, this does not fairly reflect the heterogeneous digital lives of older adults. Given the diversity and complex impacts of digital technologies on later life, there is a need to advance our understanding of partnership for digital participation across different life domains, such as health, working life, leisure, or civic life. Based on the aforementioned micro, meso and macro enablers to digital participation, we propose a community-based partnership model that connects multi-level enablers. The ultimate goal is to optimise conditions and environments so that everyone has the opportunity to participate fully in digital activities in later life. We developed a visual guide for building partnerships towards this goal (Figure 1).

A possible direction towards partnership for digital participation could be initiating multi-stakeholder collaborations around promoting the purposeful use of digital technologies. Key stakeholders include older adults, their families and friends (micro level), private sector companies, academia, media, communities and civil society organisations (meso level). Examples of macro level stakeholders are national governments and authorities.

Specific partnership goals as outlined in Figure 1 might be to leverage existing knowledge, skills, and resources. This includes engaging community stakeholders, identifying financial and non-financial resources to initiate and sustain digital participation programmes, and connecting individual needs and preferences with existing resources. Another goal is to advance the creation of digital opportunities within AFCCs. This might include improving technology usability and accessibility, but also involve older adults and relevant stakeholders in technology design, use and deployment. Opportunities might also encompass the co-production and communication of age-friendly information, where older adults can digitally take part as consulting citizens and active contributors (Reuter et al., 2020a). One example is the use of audio-visual media by older adults in the community to advocate for age-friendliness in local planning (Clarke et al., 2016). Another example is the creation of radio shows to promote diverse voices of older adults in public debate (Later Life Audio and Radio Cooperative, 2022). In these example practices different generations, municipalities and local authorities, researchers and civil society organisations are relevant stakeholders.

Another direction is to expand social networks and enhance the integration of digital and non-digital services provided by various sectors. It may take place in different forms, for example inspired by the concept of social prescription (Bertotti et al., 2018), which connects people with services. Taken further, a digital prescription approach could connect older adults beyond close contacts with multiple sectors and cover a wide range of digital services, integrated information and communication systems. To sustain these partnerships, it is important to address the sustainability of digital participation programmes for long-term impacts by reinforcing political commitment, securing funding and utilising existing resources. It is essential to amplify the innovative interventions with scale-up potential by customising approaches to unique circumstances and improving profitability. A second focus should be given to connecting generations for digital interaction as part of these partnerships. By taking on an intergenerational approach, understanding, learning and mutual respect can be promoted between generations and skills and opportunities identified.

Policymakers are advised to review and develop digital policy to ensure that issues of ageing are considered and addressed. Such efforts may incorporate later-life digital participation within policy agendas, reframing ageing and technology in policy discourse, and propose concrete measures for implementation. These efforts need to be informed by the best available evidence; it is vital to strengthen knowledge generation and translation into policy and practise. Conducting community-based or participatory action research (Corrado et al., 2020) may drive partnerships and changes for digital participation in later life. Utilising scientific expertise and knowledge traditionally held by academic institutions could support older individuals in developing digital skills in social settings (including intergenerational contexts) and benefit civic society at large. In academic endeavours, researchers play a positive and mediating role in improving later-life digital participation across levels, sectors and stakeholders. Indeed, researchers and older adults in communities could jointly induce positive changes in digital community lives.

## 4. Call to action

We call to create, develop and sustain partnership in project-based participation initiatives alongside structured or institutionalised learning schemes, with the vision of a digital society where older adults can fully participate and enhance their creative power. Such initiatives should consider online-offline hybrid participation to be inclusive and ensure that older adults’ preferences and desires are considered and achieved to the fullest extent possible. In this way, older adults will be able to engage in the partnership approach and have opportunities to enhance their digitalised participation, if they so wish. Both digital inclusion and participation approaches to improve the lives of older adults should co-exist. Specifically, digital learning opportunities should be scaled up and implemented in communities to ensure basic levels of digital inclusion and literacy. Additionally, active citizen participation in later life as well as solidarity across generations and communities should be strengthened by creating more project-or programme-based participation opportunities. Taking multi-level enablers of digital participation in later life into account, we call for multi-layered and multisectoral partnerships that optimise conditions and environments for older people’s diverse, meaningful and fulfilling engagement with ICTs.

To further implement this partnership approach, we suggest first, that communities ensure digital inclusion for all ages, promote digital participation in later life, and integrate digital participation into community development agendas. Second, multi-sectoral and stakeholder collaboration for digital participation should be strengthened within and across communities. The digital sector, especially the digital public sector (e.g., e-Government using technology to provide services to citizens), can shape digital participation in later life by raising awareness for the issue in their collaborations with other sectors. Third, we emphasise the importance of community champions in mediating communications across different levels for partnership-building. Any relevant stakeholders can take the lead on and contribute to building partnerships. Last but not least, older people should be considered as both citizens and partners in optimising environments for digital participation.

This article was written by researchers based in Sweden, which is the country with the highest levels of digitalisation in Europe. Future studies are needed to evaluate the scalability and key areas of the proposed framework and provide insight into its application in different national contexts.

## Data availability statement

The original contributions presented in the study are included in the article/supplementary material, further inquiries can be directed to the corresponding author.

## Author contributions

AR and WX: conceptualisation and writing—original draft preparation. SI, TO, and SS: review and editing. All authors contributed to the article and approved the submitted version.

## Funding

This research was funded by LMK-stiftelsen, Foundation for Interdisciplinary Scientific Research. We received funds to support the article processing fees from Lund University.

## Conflict of interest

The authors declare that the research was conducted in the absence of any commercial or financial relationships that could be construed as a potential conflict of interest.

## Publisher’s note

All claims expressed in this article are solely those of the authors and do not necessarily represent those of their affiliated organizations, or those of the publisher, the editors and the reviewers. Any product that may be evaluated in this article, or claim that may be made by its manufacturer, is not guaranteed or endorsed by the publisher.
